# Density-dependent effects are the main determinants of variation in growth dynamics between closely related bacterial strains

**DOI:** 10.1371/journal.pcbi.1010565

**Published:** 2022-10-03

**Authors:** Sabrin Hilau, Sophia Katz, Tanya Wasserman, Ruth Hershberg, Yonatan Savir

**Affiliations:** 1 Department of Physiology, Biophysics and Systems Biology, the Ruth and Bruce Rappaport Faculty of Medicine, Technion-Israel Institute of Technology, Haifa, Israel; 2 Rachel & Menachem Mendelovitch Evolutionary Processes of Mutation & Natural Selection Research Laboratory, Department of Genetics and Developmental Biology, the Ruth and Bruce Rappaport Faculty of Medicine, Technion-Israel Institute of Technology, Haifa, Israel; Pázmány Péter Catholic University: Pazmany Peter Katolikus Egyetem, HUNGARY

## Abstract

Although closely related, bacterial strains from the same species show significant diversity in their growth and death dynamics. Yet, our understanding of the relationship between the kinetic parameters that dictate these dynamics is still lacking. Here, we measured the growth and death dynamics of 11 strains of *Escherichia coli* originating from different hosts and show that the growth patterns are clustered into three major classes with typical growth rates, maximal fold change, and death rates. To infer the underlying phenotypic parameters that govern the dynamics, we developed a phenomenological mathematical model that accounts not only for growth rate and its dependence on resource availability, but also for death rates and density-dependent growth inhibition. We show that density-dependent growth is essential for capturing the variability in growth dynamics between the strains. Indeed, the main parameter determining the dynamics is the typical density at which they slow down their growth, rather than the maximal growth rate or death rate. Moreover, we show that the phenotypic landscape resides within a two-dimensional plane spanned by resource utilization efficiency, death rate, and density-dependent growth inhibition. In this phenotypic plane, we identify three clusters that correspond to the growth pattern classes. Overall, our results reveal the tradeoffs between growth parameters that constrain bacterial adaptation.

## Author summary

Even bacteria within the same strain have significant variability in their parameters that dictates their growth dynamics, such as maximal growth rate and death rate. The tradeoffs between these different parameters affect bacteria’s ability to adapt to different environments. Here, we analyzed strains of the bacteria *Escherichia coli* from different animal hosts and showed that their growth dynamics are clustered into three typical classes. To understand the underlying processes that dictate this variability, we developed a mathematical model and used it to infer the kinetic parameters of each strain. Our results show that a density-dependent growth term is crucial to explaining the experimental data. We show that the primary determinant of maximal biomass of each strain is not its maximal growth rate or death rate but rather the critical density at which growth is inhibited. We characterize the tradeoffs between the different kinetic parameters and show that the difference between the strains can be reduced to a two-dimensional plane. In this plane, the strains are divided into three main types with different kinetic parameters that define the typical growth patterns. Our results show how the tradeoffs between different growth parameters constrain growth dynamics and the ability of bacteria to adapt to different environments.

## Introduction

Adaptation of complex dynamics often involves tradeoffs between various phenotypic parameters that shape the phenotypic landscape [1, 2]. In the case of bacteria, tradeoffs between traits such as maximal growth rate, resource utilization, survival under starvation, and yield (number of doublings per resource), play a major role in their ability to adapt to different environments [3–8]. Bacterial dynamics are known to vary significantly between different bacterial species. When introduced into a new environment in which the resources are not replenished, dynamics have a few phases: after a lag phase the bacteria begin to grow exponentially and then slow to sub-exponential growth. Once bacteria reach their maximal capacity, they enter the stationary phase, where growth and death rates are comparable. The stationary phase cannot be maintained indefinitely, and so bacteria enter the death phase, during which they undergo an exponential loss of viability. During the death phase, bacteria do not die completely. A small fraction enters a Long Term Stationary Phase (LTSP) in which they manage to maintain their viability [9–13].

While the tradeoff between maximal growth and resource utilization was previously explored [7, 8], the relations between other traits that are crucial to bacterial dynamics, such as death rate and density-dependent effects, are poorly characterized. This leaves us with a limited understanding of which parameters of the growth dynamics determine bacterial fitness within the environments to which they are adapted, and the tradeoffs that underly this adaptation.

Growth and death dynamics are known to vary significantly between different bacterial species. This variability is reflected in several parameters. The most well studied so far is variation in the maximal doubling time of different bacterial species. For example, *Escherichia coli* undergoes cell replication approximately every 20 minutes, *Pseudomonas aeruginosa*, replicates about every 30 minutes, whereas *Syntrophobacter fumaroxidans* can replicate only once every 140 hours [14, 15]. Closely related strains of the same species can also display phenotypic diversity in various properties, such as sensitivity to various stresses [16], resistance to antibiotics [17], cell size and shape [18]. Indeed, there is such variation between closely related strains that underlies the adaptation of specific strains to their environments. Yet, variation in growth dynamics between closely related bacterial strains has not been well characterized.

Mathematical models are widely used for understanding and predicting bacterial behavior under different environmental conditions (such as environments that vary in their pH or temperature) [19, 20]. Bacterial growth kinetics were previously described by a large variety of mathematical models [20, 21]. While most of these models describe the first three phases of the bacterial growth curve, only a few models include the death phase and present it separately from growth [22, 23]. In contrast to parameters related to growth, parameters related to death are often neglected [24]. Death rates of various bacterial strains and species are not characterized often, at least partially due to technical difficulties resulting from the widespread use of optical density measures of cell growth, that cannot capture reductions in cell numbers for most species. As a result, the role of death rate in the tradeoffs shaping bacterial adaptation is still not well understood.

Bacterial growth is often limited by resource availability. The response of bacterial growth to resource limitation is often captured in models of growth by the Monod equation that describes the relationship between resource availability and growth rates [25]. At the same time, bacterial growth can also be limited by intrinsic mechanisms that reduce growth, once populations reach certain densities. The signal-response mechanism that enables bacteria to synchronize growth at the population level and alter their growth in response to increases in cellular density is known as quorum sensing (QS)[6, 26–28]. QS is based on the production and secretion of molecules, called autoinducers, to the medium. These autoinducers accumulate as a function of population density, reaching a minimal threshold concentration detected by the bacteria. Once this concentration is reached, bacteria alter their gene expression, leading to changes in various processes, such as biofilm formation, toxin and virulence factor production, exopolysaccharide production, motility, and growth dynamics [29, 30]. Growth rate is one of the parameters that declines drastically in response to increased density [31]. While density-dependent effects substantially affect growth dynamics, usually they are not parameterized in mathematical models of growth dynamics.

Here, we develop a novel, phenomenological mathematical model of bacterial growth dynamics that parameterizes both resource limitation and density-dependent effects and that considers all four stages of the bacterial growth curve. We apply this model to the data we collected from nine natural *E*. *coli* isolates and two *E*. *coli* lab strains and demonstrate that the model captures their growth dynamics. We show that bacterial population dynamics cannot be described well without including parameters describing *both* the density-dependent and resource-limitation-dependent effects on growth. Applying our model to data shows that variation in maximal fold change between strains is determined mainly by the cellular density at which the growth rate is reduced, rather than by the maximal growth and death rates. We also show that across *E*. *coli* strains, strains that slow down growth at higher cellular densities tend to utilize their resources more efficiently. Finally, we show that because maximal doubling time is fairly consistent across strains and due to the correlation between the density-dependent effect and resource utilization efficiency, the growth dynamics of any specific strain can be well-characterized using only its death rate and density-dependent effect.

## Results

### Different *E*. *coli* strains show significant variability in their growth and death dynamics

Various *E*. *coli* strains populate different environments that vary in their evolutionary pressures and types of stress. As a result, the strains adapt to their environment by optimizing their growth and death dynamics according to environmental demands. To obtain growth data for 11 *E*. *coli* strains originating from different environments, we performed both optical density (OD) and colony-forming units (CFU) measurements (see Methods section). OD measurements are a proxy for the total number of bacteria (live and dead) present within a sample. OD measurements enable fine-grained estimation of growth rates, but do not enable one to quantify death rates, as dead cells and their debris, continue to be measured.

CFU measurements, in contrast, enable the estimation of both growth and death rates, but can only feasibly be carried out at longer intervals.

**Fig 1:**
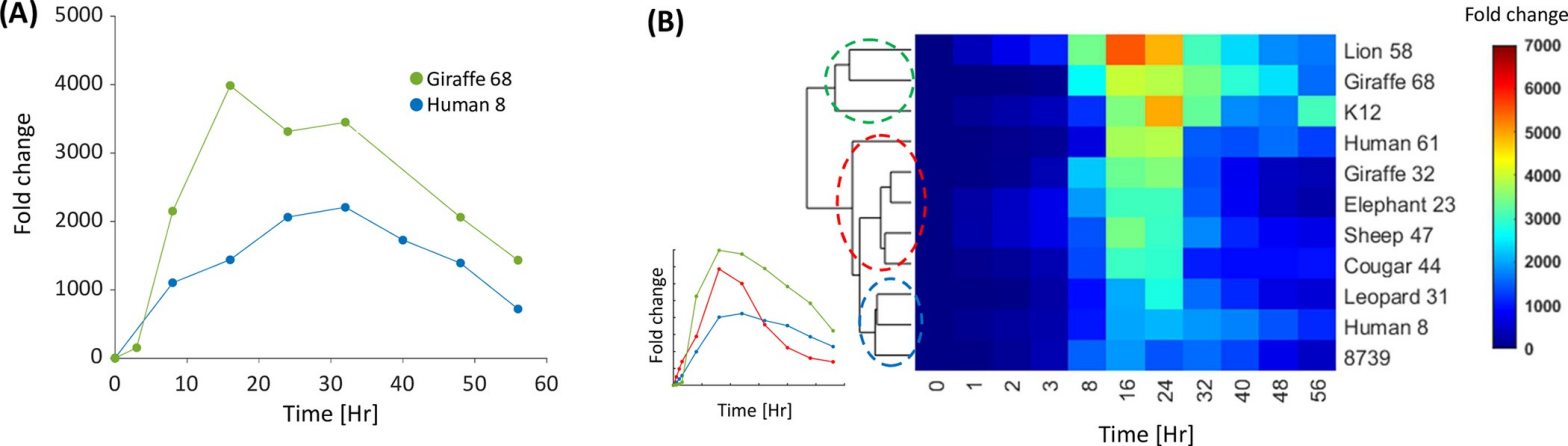
*E*. *coli* strains show significant variability in their growth and death dynamics. **(A)** Two representative growth curves of two samples from different natural strains. The dots represent the bacteria live count in a specific time point normalized by the starting point. **(B)** Hierarchical clustering of the median growth curves of 11 *E*. *Coli* strains isolated from various environments. The analysis reveals three groups that exhibit different dynamics patterns. The analyzed time series vectors are the median trajectories of 3 to 7 replicates for each time point. The inset depicts the growth trajectory of a representative replicate from each cluster.

For each strain, we measured between 3 to 7 replicates of CFU growth trajectories (S1 Fig, S1 Data). Fig 1A depicts an example for growth curves extracted, based on CFU quantification, for two of the 11 considered strains, and exemplifies the clear variation observed between strains (Fig 1A). To validate and estimate the significance of the variation observed between the growth curves of each pair of the 11 strains, we treated each curve as a time series and performed hierarchical clustering analysis on their median time trajectory (Fig 1B). The clustering reveals three main groups of strain growth curves with different dynamical properties. Similar groups emerge if the clustering is done on all the samples (S2 Fig). The first group (green, Fig 1B) is characterized by high maximal fold change (FC) and slow death, the second group (blue, Fig 1B) is characterized by low maximal FC and slow death, and the third group (red, Fig 1B) has intermediate maximal FC values and can be further divided into two groups that are differed by their death rate.

**Fig 2:**
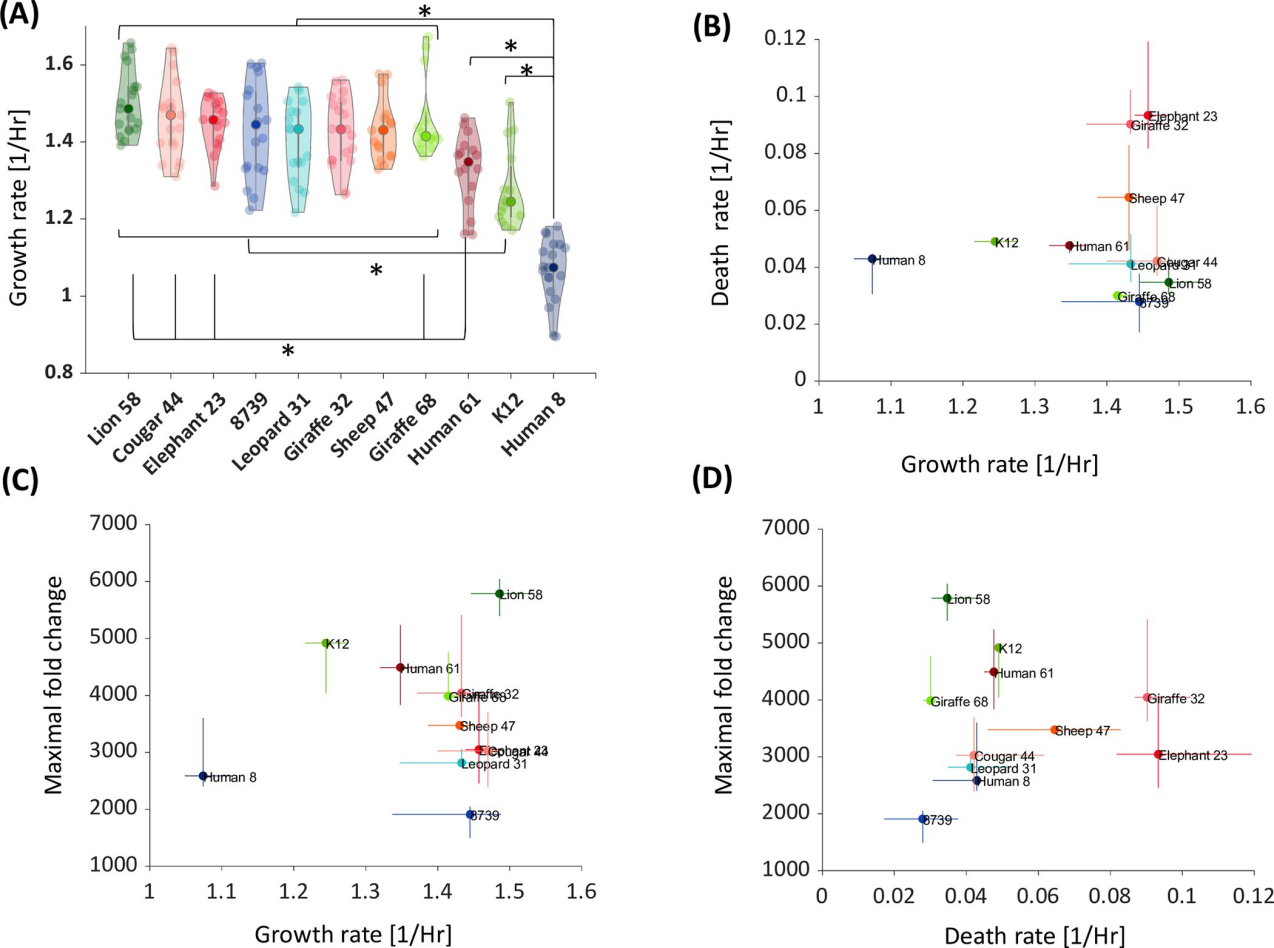
Maximal growth rate and death rate do not account for strain variability. **(A)** Violin plot of the maximal growth rate distribution. The bold dots represent the median maximal growth rate. The other dots represent the different samples. The asterisks denote distributions that are different according to the Kolmogorov-Smirnoff test with a p-value<0.05. **(B)** Death rate vs. maximal growth rate. The colored dots represent the median rates. The error bars are the 34 and 66 percentiles. Maximal growth rate and death rate have no significant correlation between them (Pearson: ρ = 0.13, p-value = 0.17). There is also no significant correlation between **(C)** Maximal fold change (FC) and growth rate (Pearson: ρ = 0.084, p-value = 0.81). **(D)** Maximal FC and death rate (Pearson: ρ = 0.011, p-value = 0.97).

To characterize the relationship between the observed maximal growth rate, death rate, and maximal FC, we derived the maximal growth rate and the death rate for each strain, from the growth curve data (see S3 and S4 Text, S1, S2 and S3 Data). A low variation in maximal growth rates was observed between strains (Fig 2A). Only two strains (Human 8 and the K12 lab strain) significantly varied from the remaining nine strains. In contrast to maximal growth rates, maximal FC and death rates varied more substantially between strains (S3 Fig). We observed no correlation between maximal growth rate and either death rates (Fig 2B), or maximal FC (Fig 2C). Finally, death rates also do not correlate significantly with maximal FC (Fig 2D).

### A mathematical model that takes only resource limitation into account cannot capture the observed variation in growth and death dynamics

**Fig 3:**
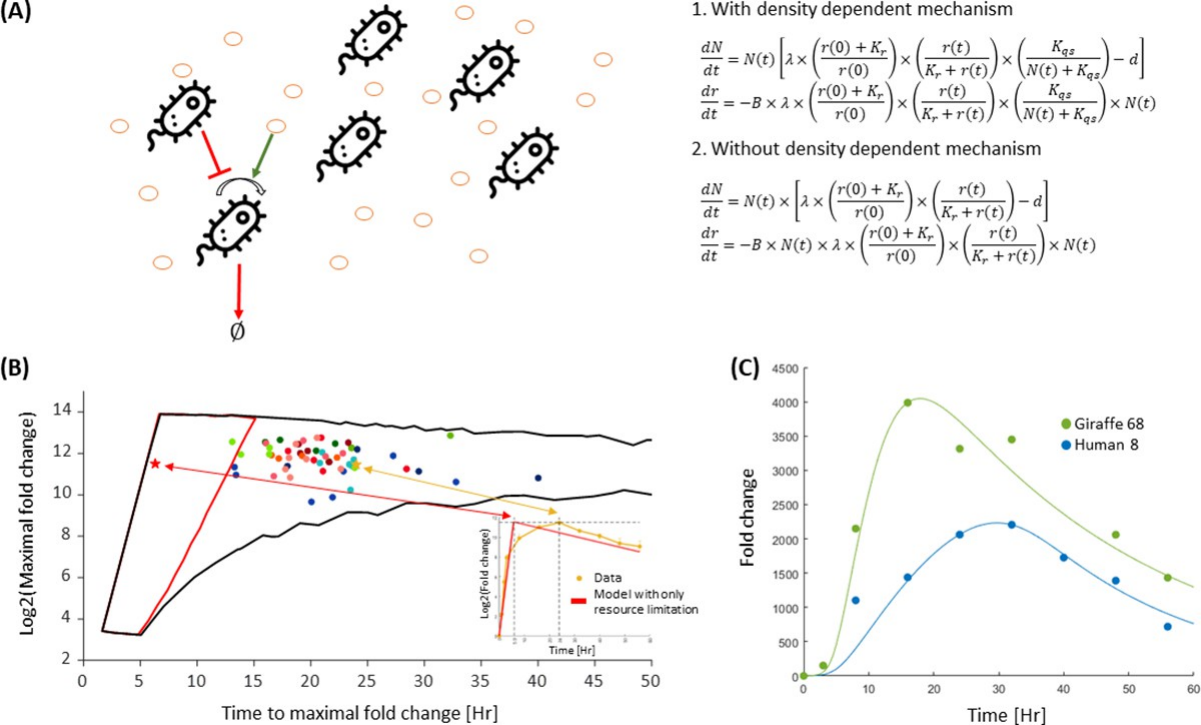
Density-dependent limitation and resource limitation are both necessary to capture the entire growth dynamics. **(A)** Growth and death dynamics regulation is composed out of two environmental factors and described by two coupled ODEs. The orange circles denote the resource. The resource is needed for bacterial growth (green arrow), and bacterial density negatively affects growth (red arrow). The first two coupled ODEs describe these dynamics and include two growth rate modulators: resource amount and bacterial density. The second pair of ODEs describe bacterial growth dynamics considering only resource limitation. (Bacterium icon made by Freepik from www.flaticon.com). **(B)** Quantifying the models’ limits. The black lines are the boundaries of the possible values of maximal FC and time to maximal FC from the model that takes both resource limitation and density-dependent (model (1)). The red lines represent the boundaries of the model that accounts only for resource limitation (model (2)). These values are estimated for wide ranges of *λ* (0.8–1.5), *B* (10^−4^–10^−3^), *K*_*r*_ (0–1) and, in the case of model (1), *K*_*qs*_ (10^2^−10^3^). The model with only resource limitation cannot capture the experimental data (full colored circles). The inset illustrates an example of an experimental measurement that is outside the resource limitation feasible space (red star). The red curve is the resource limitation model that has the same maximal FC as the data. Note that for a model that accounts only for resource limitation, the time to maximal FC, for a given maximal FC, is much shorter than the observed times. **(C)** A representative fit of the model with both resource limitation and density-dependent effect on the data. The presented samples are the two samples that were shown in Fig 1A. The fits for all the samples are shown in S5 Fig.

The Monod model, which takes into account resource limitation as a growth rate modulator, is commonly used for modeling bacterial growth[27, 32]. Following Monod, we developed a mathematical model that integrates growth, death and resource limitation (Fig 3A). The model is composed of two coupled ordinary differential equations (ODE) for the dynamics of bacteria number, *N*, and the resource, *r*,

$$\begin{array}{l} \frac{dN}{dt}=N(t)\times \left[\lambda \times (\frac{r(0)+K_{r}}{r(0)})\times (\frac{r(t)}{r(t)+K_{r}})-d\right]\\ \frac{dr}{dt}=-B\times \lambda \times \left(\frac{r(0)+K_{r}}{r(0)}\right)\times \left(\frac{r(t)}{r(t)+K_{r}}\right)\times N(t) \end{array}$$ (1)

Where λ is the maximal growth rate, *d* is the death rate, *B* is the number of resource units the bacterium needs to divide once, *r*(0) is the initial resource where there is no resource limitation, and *K*_*r*_ is the resource scale at which resource limitation is effective (in the case *K*_*r*_ <<*r*(0), *K*_*r*_ is approximately the concentrations at which the growth rate is half the maximal growth rate). We have normalized the resource value at time zero to be one, *r*(0) = 1. We find that this model cannot capture the entire observed growth and death dynamics (Fig 3B, S4 Text and S4 Fig). One of the main limitations of this model is that it predicts that a population will reach maximal FC faster than it actually does. Fig 3B illustrates the phase space of the relationship between the time to reach maximal FC and the values of the maximal FC, for a broad set of parameters. Most of the experimental observations fall outside of this regime. Thus, this model cannot capture the variation in the observed dynamics between strains, not because of poor parameter fitting, but rather due to the inherent structure of the model.

To gain an intuition for this phenomenon, it is useful to consider the simplest case in which each dividing cell is consuming *B* units of resource per division (*K*_*r*_ → 0). In this case, as long as there is more resource than the number of cells multiplied by *B*, *B*⋅*N*(*t*), all the cells can divide. When the resource is smaller than *B*⋅*N*(*t*), there is not enough resource to support the doubling and thus, in this simple case, the growth is exponential until the last doubling, and the time to the maximal fold change is determined mainly by the maximal growth rate. Increasing *K*_*r*_ can slow down because growth limitation occurs at higher resource concentrations. However, *K*_*r*_ cannot be arbitrarily high, but to be consistent with the observed exponential growth of the first few doublings. The measured dynamics, exhibit a significant sub-exponential growth phase. As the resource limitation model cannot recapitulate the transition from exponential growth to long sub-exponential phase, it yields a faster time-to-maximum than the observed.

We also consider the effect of adding a Hill coefficient to the resource limiting model (S4 Text). As expected, increasing the Hill coefficient results in a sharper transition between the exponential and sub-exponential regimes. Yet, even when adding the Hill coefficient, the resource limitation model still cannot capture the observed longer time to maximal FC (S4 Text and S4 Fig).

#### Density-dependent effects determine maximal FC variability

Since a model that considers only resource limitation cannot capture our observed growth dynamics, we added to the model another term that modulates growth in a density-dependent manner,

$$\begin{array}{l} \frac{dN}{dt}=N(t)\times \left[\lambda \times (\frac{r(0)+K_{r}}{r(0)})\times (\frac{r(t)}{r(t)+K_{r}})\times (\frac{K_{qs}}{N(t)+K_{qs}})-d\right]\\ \frac{dr}{dt}=-B\times \lambda \times \left(\frac{r(0)+K_{r}}{r(0)}\right)\times \left(\frac{r(t)}{r(t)+K_{r}}\right)\times \left(\frac{K_{qs}}{N(t)+K_{qs}}\right)\times N(t) \end{array}$$ (2)

Where *K*_*qs*_ is the bacterial density at which the density-dependent term reaches half its maximal value.

By fitting the data with the modified model, we show that the density-dependent term enables us to capture the data more accurately (Fig 3B and 3C, S5 and S6 Data, S5 Fig). We also used Akaike’s Information Criterion (AIC) and Bayesian Information Criterion (BIC). To compare between models. These metrics account for the tradeoff between the goodness of fit and the number of model parameters. The model with the density-dependent term (Eq 2) is significantly better under these metrics than the one without this term (Eq 1) (S5 Text and S6 Fig). The interplay between the resource and density-dependent effects is such that the density effect is dominant earlier in the dynamics relative to resource limitation. Therefore, this model can capture the early subexponential growth and the time to maximal FC. Since the death rate is negligible compared to the maximal growth rate, the maximal FC and the time it takes to reach it are not strongly influenced by death rates (S6 Text and S7 Fig).

**Fig 4:**
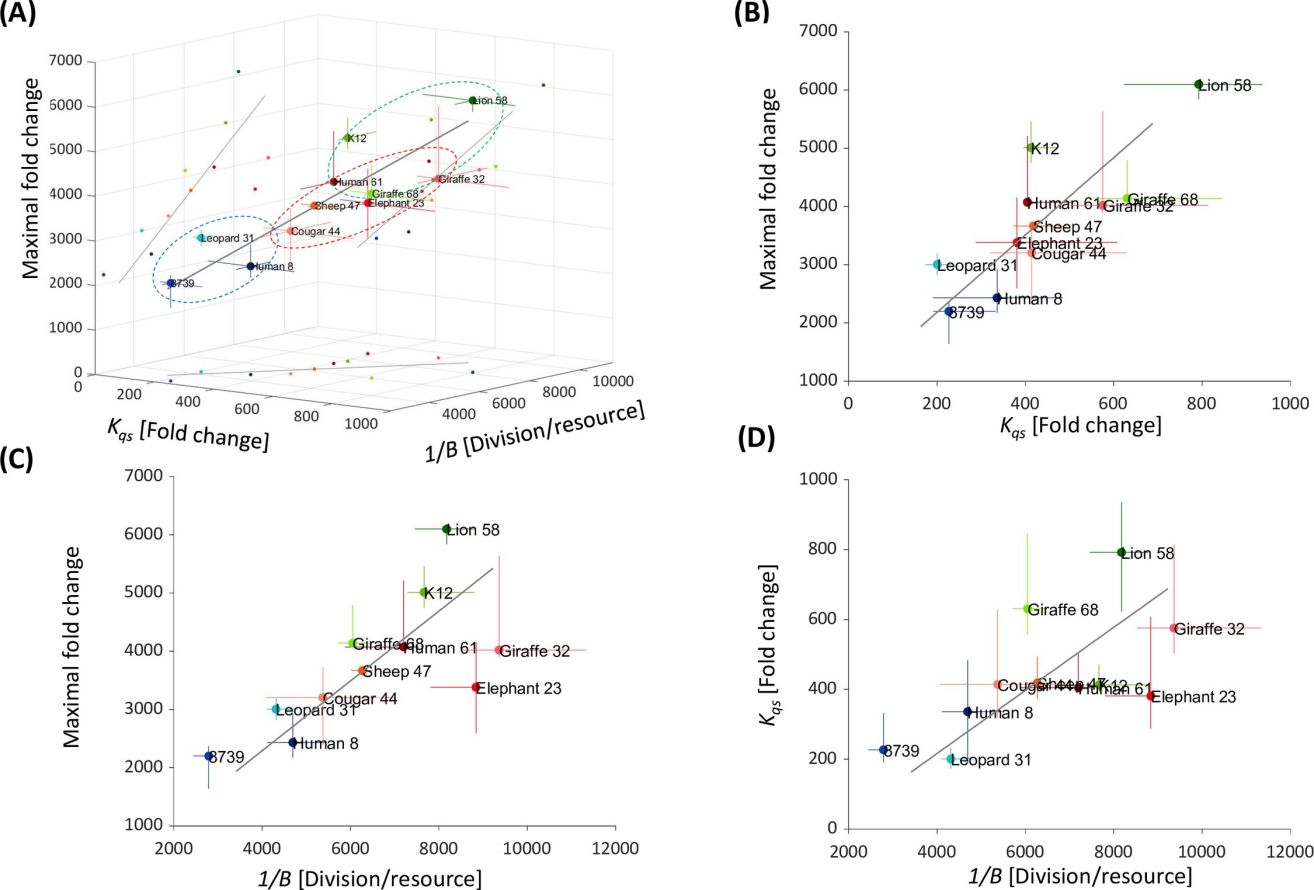
Maximal fold change is correlated with density-dependent effect and efficiency. **(A)** Maximal FC vs. *K*_qs_ and 1/*B*. The dots represent the median of each strain. The error bars are the 34 and 66 percentiles. The blue, red and green dashed-lined circles represent the group that emerged in the time series hierarchical clustering (Fig 1). The black lines are the first principal component and its projections. **(B)** Maximal FC vs. *K*_qs_. The gray line is the projection of the 3D first principal component. (Pearson: *ρ* = 0.81; p-value = 0.023). **(C)** Maximal FC vs. 1/*B* (resource utilization efficiency). The gray line is the projection of the 3D first principаl component, (Pearson: *ρ* = 0.69; p-value = 0.019). **(D)** *K*_qs_ vs.1/*B*. The gray line is the projection of the 3D first principаl component, (Pearson: *ρ* = 0.63; p-value = 0.03).

While growth rate and death rate are not correlated with maximal FC (Fig 2C and 2D) the bacterial density at which the growth rate is reduced drastically, *K*_*qs*_, and the number of divisions bacteria can undergo on a certain amount of resource units, 1/*B*, are significantly correlated with the maximal fold change and with each other (Fig 4, S4 Data). PCA analysis (Figs 4A and S8), shows that the first principal component accounts for 81.8% of the variability of these three quantities. Strains that achieve higher maximal FC, use their resources more efficiently and delay their density-dependent slowdown, relative to strains that reach lower maximal FC.

Interestingly, the groups that were identified using the hierarchical clustering of the growth curves as time series (Fig 1B) keep their structure in the phase space of these three kinetic parameters of the model (Fig 4). The blue cluster is composed out of strains that grow most slowly and have the lowest maximal FC (Fig 1B). Strains belonging to this cluster also appear to be the most inefficient in terms of resource utilization and slow their growth rate after reaching the lowest cellular density. In contrast, strains belonging to the green cluster are characterized by the highest maximal FC, are very efficient in their resource utilization and delay growth slowdown when they reach much higher densities. Strains belonging to the red cluster have intermediate maximal FC. Fitting with this, their resource utilization efficiency, and the density at which they reduce growth rates are also intermediate (Figs 1B and 4A).

### Strains reside within a two-dimensional phenotypic space

**Fig 5:**
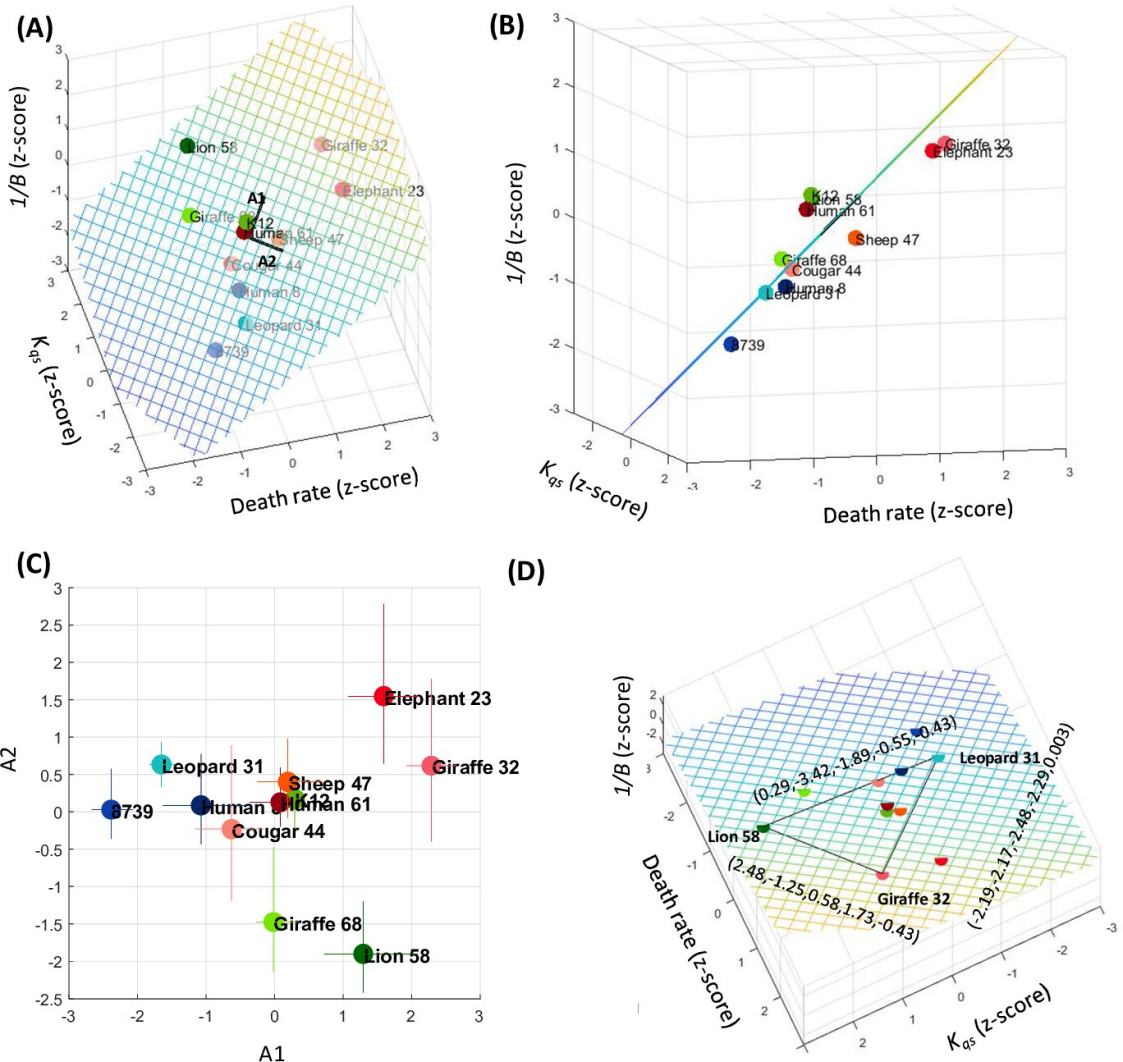
Phenotypic diversity is confined to a low dimension. **(A)** Each dot represents a strain in the three-dimensional space of *d*, *K*_*qs*_, and 1/*B*. The black vectors are the projection of the 5D first and second PCA axes, A1 and A2, respectively, to this space. The dashed plane is the 2D plane spanned by A1 and A2. The parameters reside mostly in this plane. **(B)** Projection of the data along with A1. The deviation of the data plots from the phenotypic plane is small compared to its spread within the plane. **(C)** The data projected onto the plane spanned by A1 and A2. The error bars are the propagated 34 and 66 percentiles. **(D)** The projected data in the phenotypic plane and the phenotypic distance between representative strain on the triangle’s edges (parameters order: *d*, *K*_*qs*_, 1/*B*, *K*_*r*_, *λ*).

While the model contains five parameters, the significant correlation between the density-dependent effect (*K*_qs_) on the maximal growth rate (*λ*) and resource utilization efficiency (1/*B*), suggests that the effective dimension of the phenotypic space is lower. To examine the number of parameters necessary to describe the bacterial growth and death dynamics, we performed another principal component analysis (PCA). Within this PCA, each strain was characterized by a vector composed of the five model parameters (maximal growth rate (λ), death rate (*d)*, the density-dependent effect (*K*_qs_), resource utilization efficiency (1/*B*), and the resource concentration at which resource limitation is substantial. 70.2% of the variability is explained by the first two principal axes, while >87% are explained by the first three. Fig 5 illustrates the phenotypic distribution in the death rate, efficiency and *K*_qs_ space. The data mostly reside within a two-dimensional space plane spanned by the first and second PCA axes, A1 and A2 (Fig 5A and 5B). The structure of the data within this plane is intriguing as it reveals a phenotypic distribution that is close to a triangle (Fig 5C and 5D). This type of dimensional reduction may indicate an adaptation process where the nodes of the triangles represent an archetype combining all three traits at different proportions. Each archetype is adapted to specific type of environment [2, 33] (Fig 5C and 5D).

Interestingly, each one of the different groups identified earlier, populates different nodes of the triangle. The strains on the blue edge experience a density-dependent slowdown at low densities and are inefficient in their utilization of resources. However, their death rates are the lowest. Strains on the red edge die very rapidly but are very efficient and insensitive to bacterial density. The green edge strains slow down their growth at the highest densities and are intermediate in respect to their efficiency and death rates. To validate the PCA results and demonstrate that the strains can be separated by two parameters only (*d*, *K*_*qs*,_ or 1/*B*), we evaluated the parameters that separate most significantly between the strains in the edges. To do so, we calculated the distance in the 5D phase space between the strains in the triangle nodes (Fig 5D). We observed that the edges are separated by two to three parameters, which are a combination of *d*, *K*_*qs*,_ or 1/*B*.

## Discussion

*E*. *coli* strains are part of the normal gastrointestinal tract flora. This flora is influenced by a lot of factors such as diet, stress, and medication[34]. Moreover, the bacterial content is changed along the gastrointestinal tract; every organ is characterized by different bacteria types[35]. Therefore, gastrointestinal flora could present inter- and intra-species variance. To enable us to compare the entire growth curve of different bacterial strains and to extract the parameters that influence variance between strains the most, we generated a mathematical model that fits experimentally generated growth curves. In addition to the well-explored maximal growth rate, our model allowed to extract death rates, the bacterial density at which a strain slows down its growth, *K*_*qs*_, the resource utilization efficiency,*1/B*, and the concertation of resource at which growth rates is affected by resource limitation, *K*_*r*_. This in turn, enabled us to demonstrate that maximal growth rates and death rates are not correlated and that maximal growth rates do not determine the maximal fold change. It indicates, that strains that are growing faster in their exponential phase are not necessarily reaching higher biomass or die faster.

We found that *K*_*qs*_ and *1/B* are correlated with the maximal fold change and with each other significantly. This suggests that the amount of biomass depends not on a strain’s ability to grow or die faster or slower. Instead, it depends on the cellular density at which growth is inhibited and on the efficiency with which it utilizes its resources. Intriguingly, both these parameters are strongly correlated, meaning that strains that reduce growth at higher densities, also tend to be more efficient in their resource utilization.

Density-dependent regulation of growth has been extensively studied[36, 37]. The best-characterized mechanism for density-dependent behavior regulation is Quorum Sensing (QS). Bacteria that use QS to regulate their population density produce, secrete and sense at least one type of QS molecules. The QS molecules increase as a function of bacterial density to a certain concentration threshold in which a behavioral change occurs. The tradeoffs that emerge from our results suggest that strains that limit their growth at higher densities, so they are less sensitive to QS signals, tend to utilize growth resources more efficiently and, as a result, reach higher maximal yields. This may suggest a potential cost for QS strains that start to execute QS at lower bacterial densities may need to utilize more of their resources to generate more of the signaling molecules involved in QS, leading to their lower efficiency and lower maximal FC. Fitting with this idea, it was suggested that due to metabolic tradeoff between bacterial growth and signal production and the accumulation of toxic products, signal production leads to a two-fold metabolic burden under resource limitation[38]. Inversely, it is also possible that bacteria that use their resource inefficiently for other, genetic or environmental reasons, may adopt high sensitivity to population density, in order to better regulate their growth and protect themselves from extinction. In an environment where the resource is scarce, the effect of density-dependence could be more limited. Interestingly, the density-dependent term is already effective even after two or three doublings (S5 Fig). That is, even in environments that support only a few divisions, density dependence plays a role.

**Fig 6:**
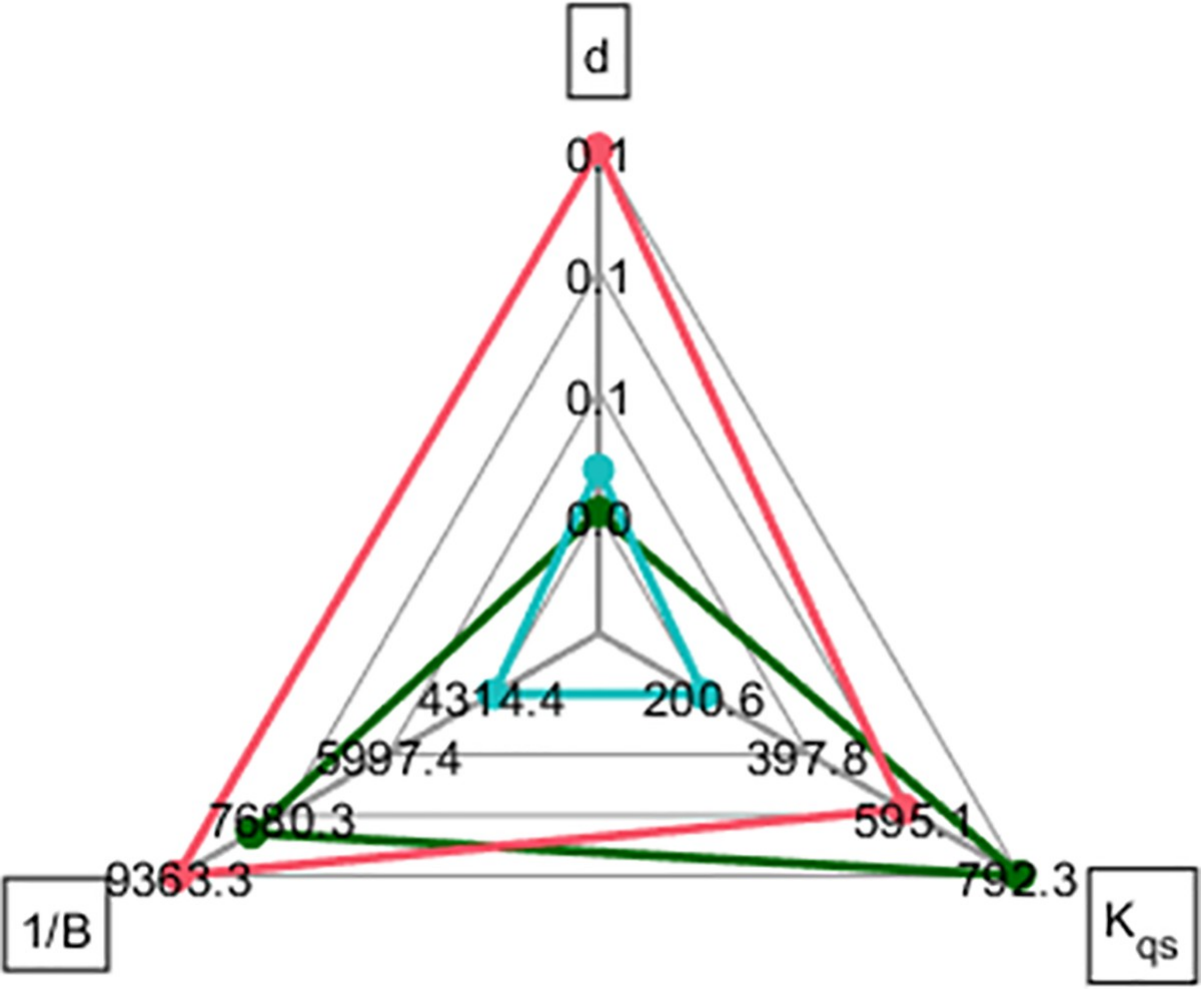
Spider plot for representative strains. Giraffe 32 (red), Lion 58 (green) Leopard 31 (cyan).

Our analysis shows that the phenotypic landscape resides within a two-dimensional subspace of the five-dimensional parameter space. Specifically, it is spanned by the density-dependent effect (*K*_*qs*_), resource utilization efficiency (1/*B*, which is strongly correlated with *K*_*qs*_), and death rate (*d*). Moreover, the phenotypic distribution within this plane takes the shape of a triangle. This type of structure was suggested to reflect the result of adaption under constraints [1, 2] such that each triangle node represents a desired bacterial “archetype”. Remarkably, the strains on the nodes of this triangle correspond with the clusters that were identified in our time-series based clustering (Fig 1), and similar to the clusters revealed in the maximal FC-*K*_*qs*_-1/*B* phase space (Figs 4A, 5 and 6). Members of the first archetype (the blue cluster) can be seen as “inefficient growers /strong communicators”. Their density-dependent growth slowdown occurs at low bacterial concentration (meaning they likely respond strongly to QS), and their efficiency is low. Death rates within this cluster are low. Members of the second archetype (the red cluster), are highly efficient, but die rapidly. In the third archetype (the green cluster), density-dependent growth slows down at much higher cellular densities than the other archetypes. Green cluster members are also highly efficient, albeit a little less than members of the red cluster. Thus, the green archetype members are inverse to the blue archetype, in that they are “efficient growers / poor communicators”.

Interestingly, although the human strains have significantly different maximal fold change, they exhibit proximity in the reduced phenotypic landscape of the archetypes, suggesting that their bacterial species are influenced by a common environmental or physiological characteristic. It is insightful to consider the possible relation between the archetype and the environment. In a poor resource environment, there is no advantage in limiting growth, not by density limitation nor by death. In this type of environment, strains would benefit from a high critical density and low death rate. This will enable bacteria to grow exponentially upon nutrient availability, without reaching the density restriction, and when reaching the point of nutrient depletion, they have the advantage of a slow death rate. Our results may suggest that the “green” strain, which exhibits high *K*_qs_ and a low death rate, will succeed in such an environment. In contrast, in a rich nutrient environment, a mechanism for avoiding bacteria overpopulation could present an advantage. There are two limiting strategies for restricting the population: low critical density that slows down growth or elevated death rate. The difference between these two strategies is in their dynamics. Strains with high growth and high death are more responsive to changes compared to strains with low growth and low death. Therefore, our results suggest that the “blue” strain which has low *K*_qs_ and low death rate would benefit from a slowly changing rich environment, while the “red” strain which has a high death rate and higher *K*_qs_, would benefit from a rich environment that is changing faster.

The archetypes we found here emphasize the tradeoffs between the three traits and demonstrate that bacteria adapt to their environment by evolving different combinations of these trait values. Thus, our results reveal that the main modulators of the *E*.*coli* fitness may be the tradeoffs between the allocation of resources to growth, its degree of interaction with its surrounding bacteria, and death rates.

## Methods

### Strains

In this study, we used 11 strains of *E*. *coli* (S1 Text, S1 Table). Nine of them are natural isolates obtained from zoo animals, or from the body of human hosts, as described in [39] and in S1 Text. The remaining two strains are laboratory strains, K12 MG 1655 and 8739.

### CFU measurements

Frozen samples were streaked on Luria-Broth (L.B.) agar Petri dishes and were incubated at 37°C overnight. Colonies were inserted into 4 ml L.B. in 15 ml test tube and were grown at 37°C with shaking at 225 rpm for two hours to mid-exponential stage. Samples were diluted to a concentration of 10^6^ per 2ml at a 12-well plate. Only eight wells from the plate were used for the experiment. The strains were cultured for 56 hours, and samples were taken at constant time points (at time zero,3 hours, 8 hours, and every 8 hours) for a total of 9-time points. We also performed experiments with finer time resolution at which sampling was performed every hour (at time zero, 1 hours, 2 hours, and every 3 hours). That is, we sampled 11 time points in total. Samples were streaked by the robot on Petri dishes containing L.B. agar. The dishes were incubated at 37°C overnight, followed by a colony count. For each strain, between 3 to 7 replicates were measured (S1 Fig and S1 Data).

### OD measurements

Frozen samples were streaked on Luria- Broth (L.B.) agar Petri dishes and were incubated at 37°C overnight. Colonies were inserted at 4 ml L.B. in 15 ml test tube and were grown at 37°C with shaking at 225 rpm for two hours to mid-exponential stage. Samples were washed twice and were diluted to 200 μl OD of 0.05 in 96-wells plate. The plate was incubated in a plate reader for 16 hours at 37°C with orbital shaking. OD was measured every 10 minutes at 600 nm. The experiment was conducted five times for each strain. Each experiment includes 3–4 samples for each strain. The OD measurements were corrected according to the plate reader calibration curve (S2 Text, S2 Data).

### Time series trajectories hierarchical clustering

Clustergram was generated by Matlab ‘clustergram’ function using squared Euclidean distance metric and average linkage method (Figs 1B and S2).

### Mathematical models optimization and parameters evaluation

In all the models, to perform the fits and to obtain the parameters that describe the strain dynamics, we took a non-linear least-square approach using the Trust-Region algorithm in Matlab. First, we found the maximal growth rates and death rates by taking only the exponential growth and decay, respectively (S2 and S3 Texts, S3 Data). The full CFU fold change dynamics (Eqs (1) and (2)) were solved numerically using “ode45” Matlab ODE solver. The initial resource concentration and the initial bacteria amount were normalized to one. 95% confidence intervals, the number of iterations to convergence, and Spearman correlation between the models and the data are shown in S5 and S6 Data. To quantitatively compare between the model described by Eq (1) and the model described by Eq (2), we used Akaike’s information Criterion (AIC) and Bayesian information Criterion (BIC) (S5 Text, S6 Fig). All the calculations and the plots were generated using Matlab.

## Supporting information

S1 Text Strains. (PDF)

S2 Text Maximal growth rate evaluation. (PDF)

S3 Text Death rate evaluation. (PDF)

S4 Text Resource dependent model with Hill coefficient. (PDF)

S5 Text Model comparison using information criteria. (PDF)

S6 Text Temporal behavior of the different growth terms. (PDF)

S1 Table Strains table. (PDF)

S1 Fig CFU fold change replicates. For each strain, we have measured between 3 and 7 replicates of the growth curve (gray lines). The median trajectory is shown in black.
(TIF)

S2 Fig Hierarchical clustering of all growth curves. The colors denote the groups according to the median dynamics clustering. The dotted lines are the clusters that emerge when all the samples are included (without adjustment of the different number of samples per strain). Most of the samples are assigned according to their median trajectory clustering (Macro accuracy of 0.77 and Macro F1 of 0.66).
(TIF)

S3 Fig Variability of maximal fold change, maximal growth rate, and death rate, and the lack of correlations among them. Violin plot of the death rate (A) and maximal fold change (B) distribution. The bold dots represent the median value for each strain. The other dots represent the different samples. The asterisks denote distributions that are different according to the Kolmogorov-Smirnoff test with a p-value<0.05. The order of the strains on the x-axis is the same as in Fig 2A. The coefficients of variation for the entire sample are 0.11, 0.62, and 0.44 for maximal growth rate, death rate, and maximal fold change, respectively. The coefficients of variation among the median of each strain are 0.09, 0.53 and 0.3 for maximal growth rate, death rate, and maximal fold change, respectively. (C) Death rate vs. maximal growth rate. (D) Maximal fold change vs. maximal growth rate. (E) Maximal fold change vs. death rate. The center of each error bar is the median value, and the error bars are the 34 and 66 percentiles. The growth rate was measured using OD, while the maximal fold and death rate were measured using CFU. Thus, in C and D, the spread of samples is shown around the medians. In E, each sample represents a simultaneous measurement of the maximal fold change and death rate.
(TIF)

S4 Fig The effect of the Hill coefficient on resource limitation model. The black and red lines are the boundaries of the possible values of maximal FC and time to maximal FC from the model with and without -dependence, respectively, as described in Fig 1 of the main text. These values are estimated for a wide range of *λ* (0.8–1.5), *B* (10^−5^–10^−3^), *K*_*r*_ (0–1), and, *K*_*qs*_ (10^2^−10^5^). The solid gray circles are the experimental measurements. The open black circles represent different values of *H* (0.25–20). The blue lines connect values with similar *H* (5, 10, 20). The green lines connect values with similar *K*_*r*_ (0.33, 0.66, 0.88, 0.997), where lighter green corresponds to smaller *K*_*r*_ value.
(TIF)

S5 Fig Overlay of the data with the model. Within each strain, the dots are measured data, and the lines are the best fit of the model that includes resource-dependent growth and density-dependent growth (Eq (2) in the manuscript). Each replicate has a different color. The Spearman correlation and p-values, together with the 95% confidence levels of the kinetic parameters for each replicate are shown in S5 Data. Most of the samples have a Spearman correlation higher than 0.85 with a p-value that is lower than 0.05.
(TIF)

S6 Fig Model comparison using information criteria. **Mean and standard deviation of the difference between the information criterion of the models with or without density-dependent term over all samples.** P-values are 2.21×10^−4^ and 4.79×10^−4^ for AIC and BIC, respectively, using Kolmogorov-Smirnov test.
(TIF)

S7 Fig The growth rate terms and death rate as a function of time. A typical realization of the change in growth terms with time. The growth terms that depend on density (purple) and on resource decline (yellow) with time. The density-dependent term affects first and is responsible for the initial, slow decline in the overall growth rate. The resource limitation term declines fast, as expected. Both growth terms affect the growth in the range where the death term is negligible.
(TIF)

S8 Fig *Kqs*, 1/*B*, and maximal fold change at the sample level. **(A)** Maximal FC vs. *K*_qs_ and 1/*B*. The dots are the parameters of individual measurement, the color denotes the strain. The black lines are the first principal component and its projections. **(B)** Maximal FC vs. *K*_qs_. The gray line is the projection of the 3D first principal component. (Pearson: *ρ* = 0.71; p-value<0.01). **(C)** Maximal FC vs. 1/*B* (resource utilization efficiency). The gray line is the projection of the 3D first principal component, (Pearson: *ρ* = 0.86; p-value <0.01). **(D)** *K*_qs_ vs.1/*B*. The gray line is the projection of the 3D first principal component, (Pearson: *ρ* = 0.602; p-value <0.01).
(TIF)

S1 Data CFU measurements. (XLSX)

S2 Data OD measurements. (XLSX)

S3 Data Maximal growth and death rates. (XLSX)

S4 Data Kinetic parameters values. (XLSX)

S5 Data Data and fit results, model convergence, confidence intervals to model with QS. (XLSX)

S6 Data Data and fit results, model convergence, confidence intervals to model without QS. (XLSX)
