# Supplementary material for: Density-dependent effects are the main determinants of variation in growth dynamics between closely related bacterial strains
Source: PLoS Comput Biol. 2022 Oct 3;18(10):e1010565. doi: 10.1371/journal.pcbi.1010565 (PMC9578580; doi:10.1371/journal.pcbi.1010565)
Supplement: S1 Text — (PDF) [file pcbi.1010565.s001.pdf]

S1 Text

Strains

In this study, we used 11 strains of *E. Coli* (S1 Table). Nine of them are natural isolates obtained, as described by Hochman and Selander [1]. We annotated the strains in this work according to their name and their number in the original reference list. The remaining two strains are laboratory strains.

References

1. Ochman H, Selander RK. Standard Reference Strains of Escherichia coli from. J Bacteriol. 1984;157: 690–693. doi:10.1152/jn.01312.2007
