# Supplementary material for: Density-dependent effects are the main determinants of variation in growth dynamics between closely related bacterial strains
Source: PLoS Comput Biol. 2022 Oct 3;18(10):e1010565. doi: 10.1371/journal.pcbi.1010565 (PMC9578580; doi:10.1371/journal.pcbi.1010565)
Supplement: S2 Text — (PDF) [file pcbi.1010565.s002.pdf]

S2 Text

Maximal growth rate evaluation

Frozen samples were streaked on Luria- Broth (LB) agar Petri dishes and were incubated at 37°C overnight. Colonies were grown to mid exponential stage (2 hours), in 4 ml LB within 15 ml test tubes, at 37°C with shaking at 225 rpm. Samples were then washed twice and diluted to 200 μl, OD of 0.05 in 96-wells plates. The plates were incubated in a plate reader for 16 hours at 37°C with orbital shaking. OD was measured every 10 minutes at a wavelength of 595 nm. The experiment was conducted five times for each strain. Each experiment included 3-4 samples for each strain.

To account for the nonlinearity of the OD measurements, the OD measurements were corrected according to the plate reader calibration curve. Using a plate with bacteria serial dilution, OD vs. density were measured. The resulted curve was fitted to a Michaelian function and was used to calibrate OD values,

(1) $$OD - Blank = \frac{\alpha \times C}{C + K}$$

where, α = 0.927 and K = 1.174.

The first two hours of the growth curve for each sample were fitted with an exponential growth equation,

(2) $$c(t) = c(0)e^{\lambda t}.$$

The λ values and their confidence levels were calculated from the fit (Fig. 2 in the manuscript).
