# Supplementary material for: Density-dependent effects are the main determinants of variation in growth dynamics between closely related bacterial strains
Source: PLoS Comput Biol. 2022 Oct 3;18(10):e1010565. doi: 10.1371/journal.pcbi.1010565 (PMC9578580; doi:10.1371/journal.pcbi.1010565)
Supplement: S3 Text — (PDF) [file pcbi.1010565.s003.pdf]

S3 Text

Death rate evaluation

The CFU data were fitted to exponential decay,

(1) $$N(t) = N(t_{\max})e^{-d(t-t_{\max})},$$

where, $N(t_{\max})$ is the maximal CFU, and *d* is the death rate. The *d* values and their confidence levels were calculated from the fit (Fig. 2 in the manuscript).
