# Supplementary material for: Density-dependent effects are the main determinants of variation in growth dynamics between closely related bacterial strains
Source: PLoS Comput Biol. 2022 Oct 3;18(10):e1010565. doi: 10.1371/journal.pcbi.1010565 (PMC9578580; doi:10.1371/journal.pcbi.1010565)
Supplement: S4 Text — (PDF) [file pcbi.1010565.s004.pdf]

S4 Text

Resource-dependent model with Hill coefficient

In this case the dynamics are described by:

(1)
$$\begin{aligned}\frac{dN}{dt} &= N(t)\times\left[\lambda\times\left(\frac{r^H(0)+K_r{}^H}{r^H(0)}\right)\times\left(\frac{r^H(t)}{r^H(t)+K_r{}^H}\right)-d\right] \\ \frac{dr}{dt} &= -B\times\lambda\times\left(\frac{r^H(0)+K_r{}^H}{r^H(0)}\right)\times\left(\frac{r^H(t)}{r^H(t)+K_r{}^H}\right)\times N(t)\end{aligned},$$

where λ is the maximal growth rate, *d* is the death rate, *B* is the number of resource units the bacterium needs to divide once, *r*(0) is the initial resource where there is no resource limitation, and K_r is the resource scale at which resource limitation is effective (in the case K_r $<<r(0)$, K_r is approximately the concentration at which the growth rate is half the maximal growth rate), and *H* is the Hill coefficient. S4 Fig depicts the values of maximal fold change and time to maximal fold changes that can be captured by the different Hill coefficients. As the Hill coefficient is larger, the transition between the maximal growth and the resource limited growth is sharper. The effect of these sharper transition is that the maximal fold change decreases. However, the effect of on the time to reach the maximal yield is non-monotonic as a function of the Hill-coefficient. As the $K_r << r(0)$, the growth limitation occurs at later stages and many division occur in the exponential growth regime. As the K_r is increased, growth inhibition starts at earlier stages and both the maximal fold and the time to reach the maximal fold decreases. At some stage increasing K_r even more results in a very early growth inhibition such the growth halt occurs faster and both the maximal fold the time to reach the maximal fold decreases. Overall, even introducing Hill dependence the resource limitation model cannot capture the observed longer time to maximal fold change.
