# Supplementary material for: Density-dependent effects are the main determinants of variation in growth dynamics between closely related bacterial strains
Source: PLoS Comput Biol. 2022 Oct 3;18(10):e1010565. doi: 10.1371/journal.pcbi.1010565 (PMC9578580; doi:10.1371/journal.pcbi.1010565)
Supplement: S5 Text — (PDF) [file pcbi.1010565.s005.pdf]

S5 Text

Model comparison using information criteria

To further characterize the dynamics model, we compared the suitability of the model with and without the density-dependent growth terms (Eq. (2) and (1) in the manuscript, respectively). We used the two most popular measures, Akaike information criterion (AIC) and Bayesian information criterion (BIC) [1]. These metrics aim to account for the best balances between the goodness of the fit and the number of parameters [2].

In both cases, the chosen IC is calculated for each model, and the one that gets the lowest value is the best model. BIC differs from AIC only by its larger penalty on the number of parameters [1].

For a non-linear model calculated with least square regression method, the criteria equations are:

(1) $$AIC = N\ln(MSE) + 2K,$$

and,

(2) $$BIC = N\ln(MSE) + K\ln(N),$$

where, N is the sample size, K is the number of parameters and MSE is the mean squared error [1]. The mean AIC with and without density-dependent term are 111.46 and 125.97, respectively. The mean BIC with and without density-dependent term is 112.18 and 126.55, respectively. S6 Fig illustrates the mean and standard deviation of the difference in information criteria between the models on the different samples.

References

1. Gu Y, Wei HL, Balikhin MM. Nonlinear predictive model selection and model averaging using information criteria. Syst Sci Control Eng. 2018;6: 319–328. doi:10.1080/21642583.2018.1496042

2. Nakamura T, Judd K, Mees AI, Small M. A Comparative study of information criteria for model selection. Int J Bifurc Chaos. 2006;16: 2153–2175. doi:10.1142/S0218127406015982
