# Supplementary material for: Density-dependent effects are the main determinants of variation in growth dynamics between closely related bacterial strains
Source: PLoS Comput Biol. 2022 Oct 3;18(10):e1010565. doi: 10.1371/journal.pcbi.1010565 (PMC9578580; doi:10.1371/journal.pcbi.1010565)
Supplement: S6 Text — (PDF) [file pcbi.1010565.s006.pdf]

S6 Text

Temporal behavior of the different growth terms

Our model contains two terms that modulate the growth (Eq. (2) in the manuscript). One growth term is modulated by resource availability and the other growth term is modulated by the bacterial density. S7 Fig shows the typical behavior of these terms as a function of time. The main effect that determines the overall growth rate is the density effect. The decline of the resources dependent term is sharp, as expected. Both effects shape the growth before the death rate becomes non-negligible.
