# Supplementary material for: Density-dependent effects are the main determinants of variation in growth dynamics between closely related bacterial strains
Source: PLoS Comput Biol. 2022 Oct 3;18(10):e1010565. doi: 10.1371/journal.pcbi.1010565 (PMC9578580; doi:10.1371/journal.pcbi.1010565)
Supplement: S1 Table — (PDF) [file pcbi.1010565.s007.pdf]

Strain name	Host	Number in reference list	Origin
**RM77C**	Human (Female)	8	Iowa
**RM183E**	Elephant	23	Washington (zoo)
**RM12**	Leopard	31	Washington (zoo)
**RM28**	Giraffe	32	Washington (zoo)
**RM1891**	Cougar	44	Washington (zoo)
**RM211C**	Sheep	47	New Guinea
**RM185S**	Lion	58	Washington (zoo)
**FN23**	Human (Female)	61	Sweden
**RM224H**	Giraffe	68	Washington (zoo)
**K12 MG 1655**	Laboratory strain	-	-
**8739**	Laboratory strain	-	-
