# Supplementary figures and images for: Density-dependent effects are the main determinants of variation in growth dynamics between closely related bacterial strains
Source: PLoS Comput Biol. 2022 Oct 3;18(10):e1010565. doi: 10.1371/journal.pcbi.1010565 (PMC9578580; doi:10.1371/journal.pcbi.1010565)

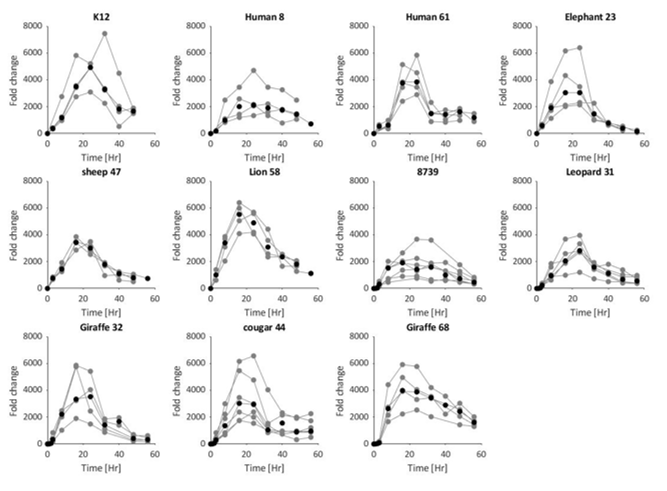

Supplement: S1 Fig — For each strain, we have measured between 3 and 7 replicates of the growth curve (gray lines). The median trajectory is shown in black. (TIF) [file pcbi.1010565.s008.tif]

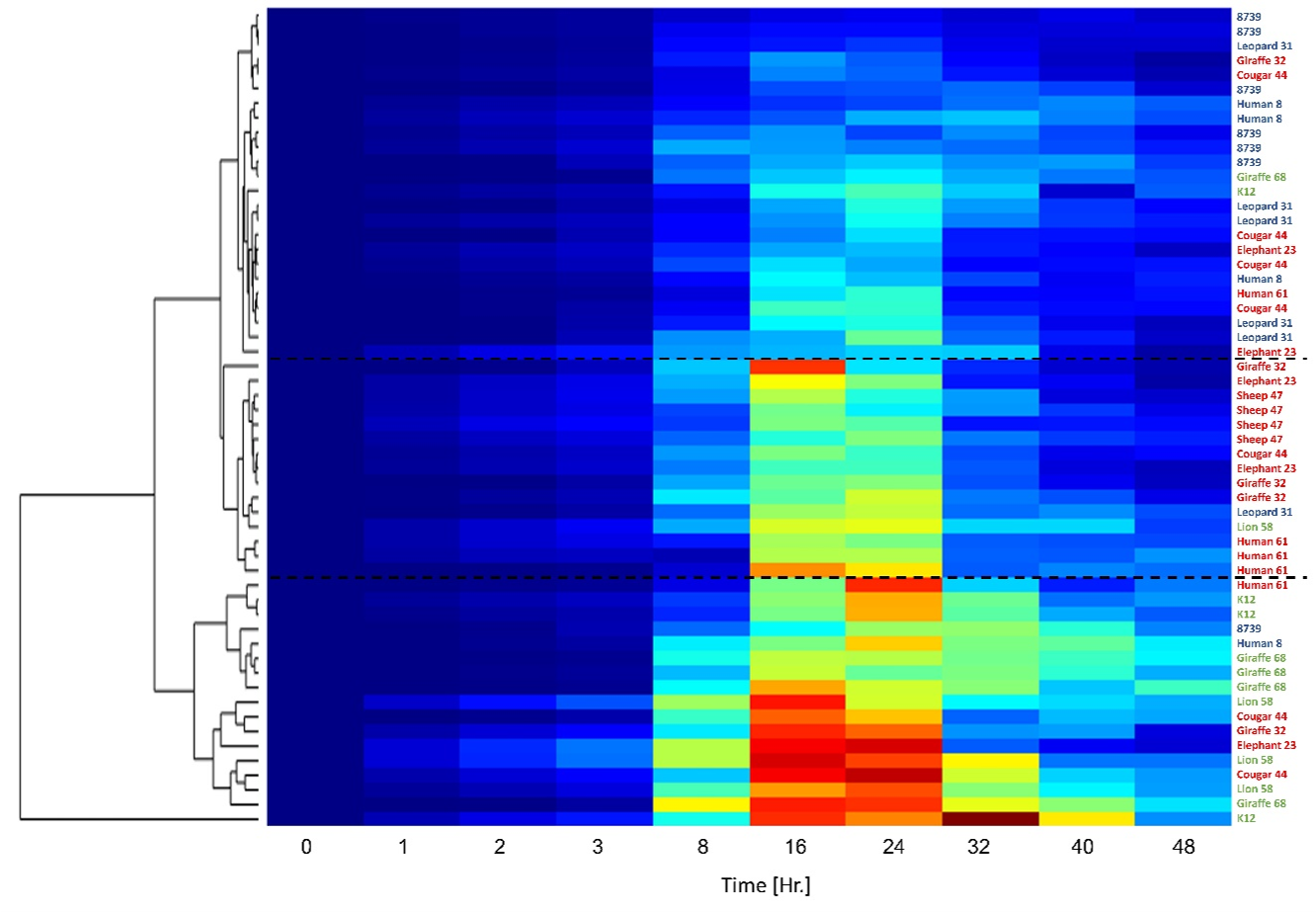

Supplement: S2 Fig — The colors denote the groups according to the median dynamics clustering. The dotted lines are the clusters that emerge when all the samples are included (without adjustment of the different number of samples per strain). Most of the samples are assigned according to their median trajectory clustering (Macro accuracy of 0.77 and Macro F1 of 0.66). (TIF) [file pcbi.1010565.s009.tif]

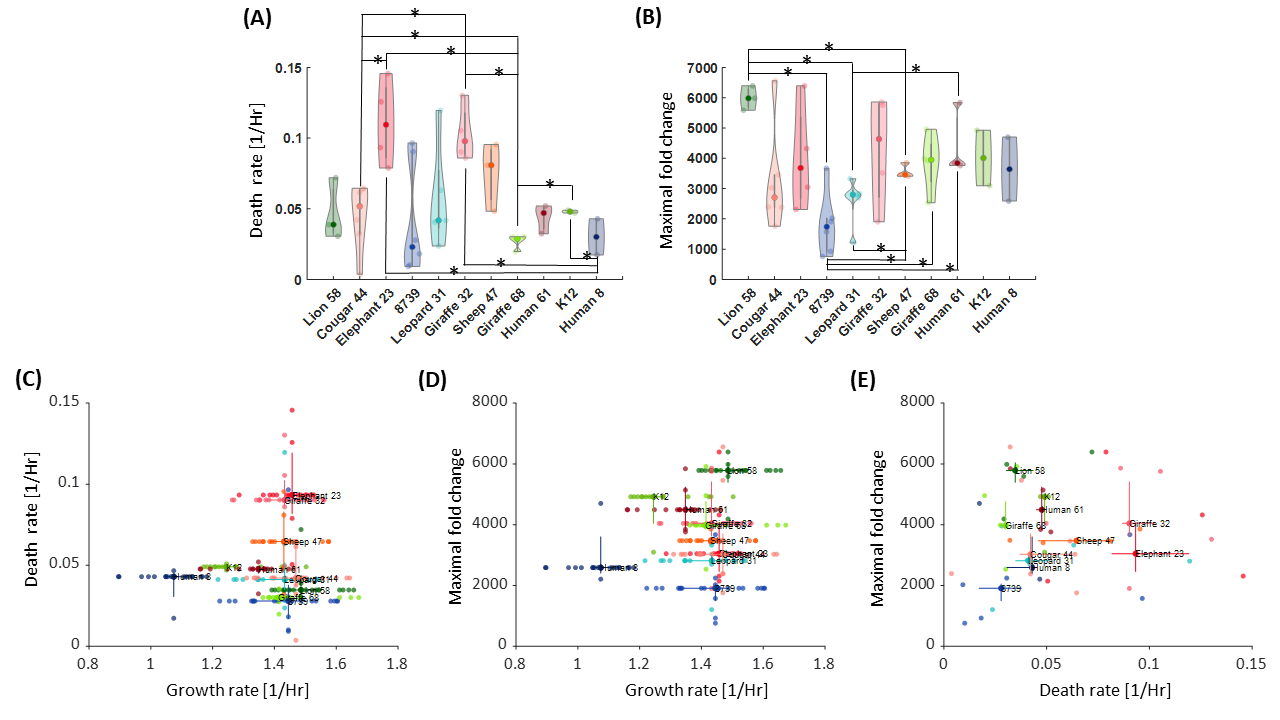

Supplement: S3 Fig — Violin plot of the death rate (A) and maximal fold change (B) distribution. The bold dots represent the median value for each strain. The other dots represent the different samples. The asterisks denote distributions that are different according to the Kolmogorov-Smirnoff test with a p-value<0.05. The order of the strains on the x-axis is the same as in Fig 2A. The coefficients of variation for the entire sample are 0.11, 0.62, and 0.44 for maximal growth rate, death rate, and maximal fold change, respectively. The coefficients of variation among the median of each strain are 0.09, 0.53 and 0.3 for maximal growth rate, death rate, and maximal fold change, respectively. (C) Death rate vs. maximal growth rate. (D) Maximal fold change vs. maximal growth rate. (E) Maximal fold change vs. death rate. The center of each error bar is the median value, and the error bars are the 34 and 66 percentiles. The growth rate was measured using OD, while the maximal fold and death rate were measured using CFU. Thus, in C and D, the spread of samples is shown around the medians. In E, each sample represents a simultaneous measurement of the maximal fold change and death rate. (TIF) [file pcbi.1010565.s010.tif]

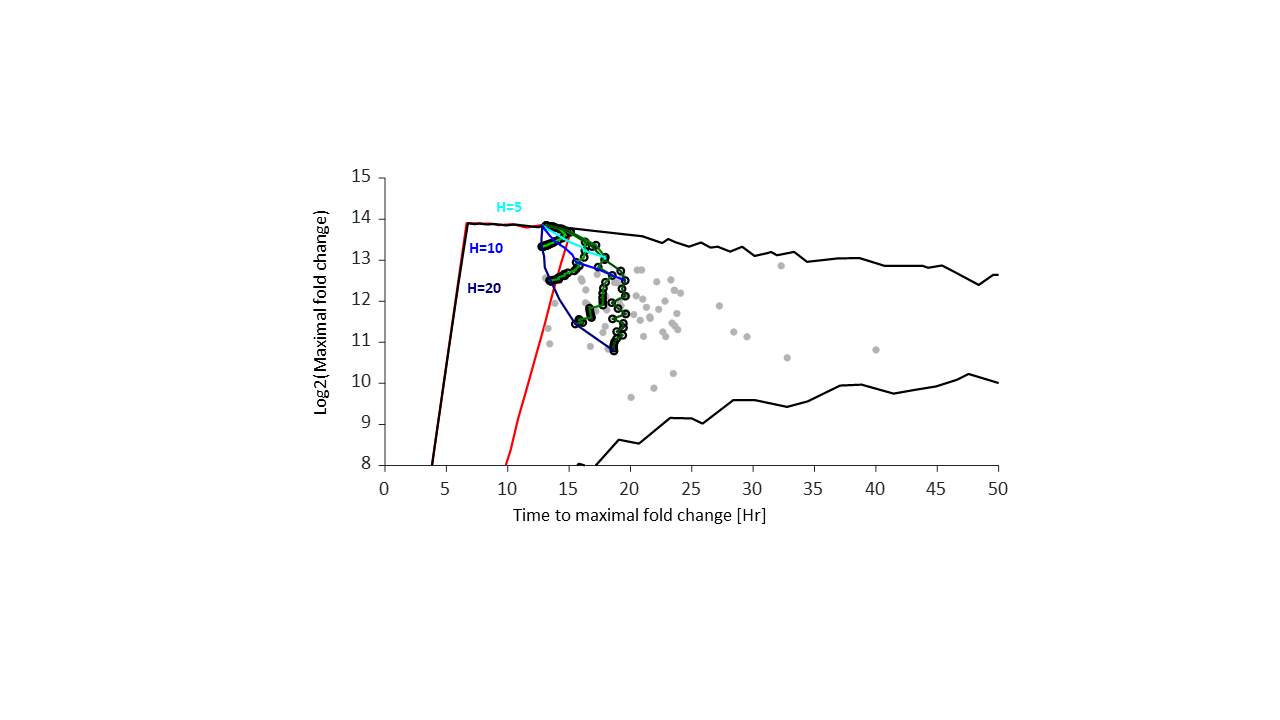

Supplement: S4 Fig — The black and red lines are the boundaries of the possible values of maximal FC and time to maximal FC from the model with and without -dependence, respectively, as described in Fig 1 of the main text. These values are estimated for a wide range of λ (0.8–1.5), B (10−5–10−3), Kr (0–1), and, Kqs (102−105). The solid gray circles are the experimental measurements. The open black circles represent different values of H (0.25–20). The blue lines connect values with similar H (5, 10, 20). The green lines connect values with similar Kr (0.33, 0.66, 0.88, 0.997), where lighter green corresponds to smaller Kr value. (TIF) [file pcbi.1010565.s011.tif]

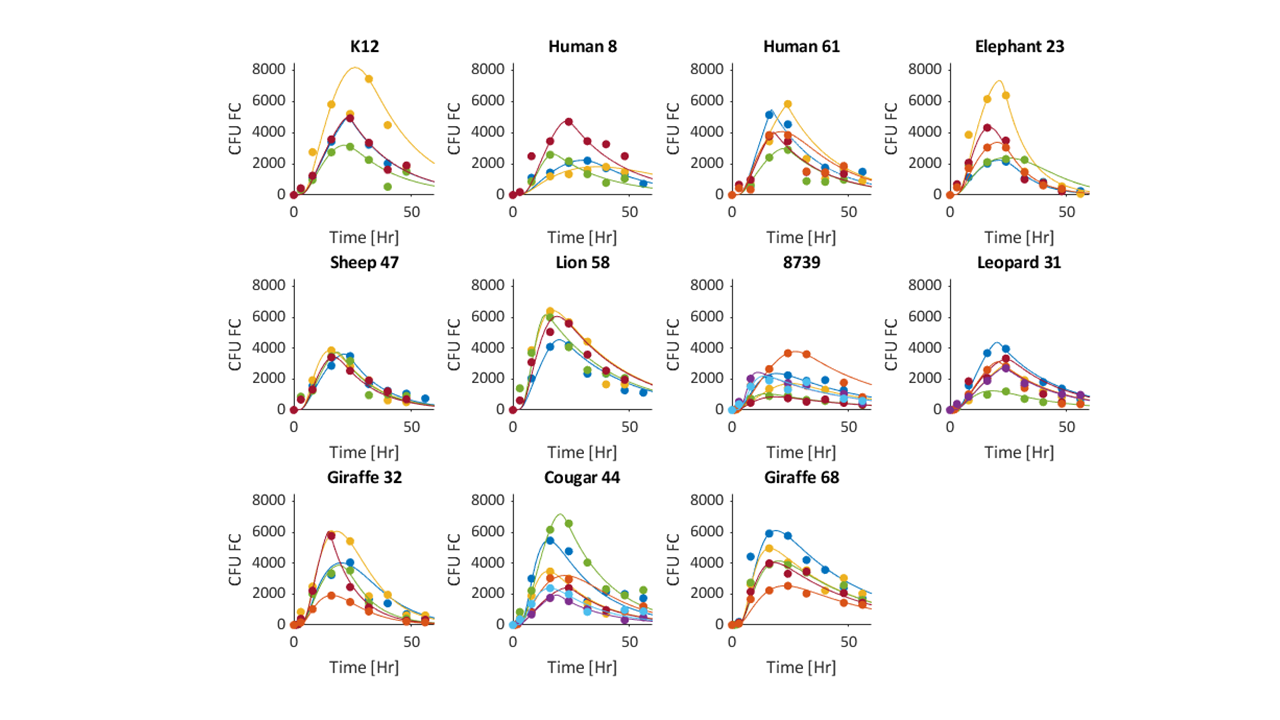

Supplement: S5 Fig — Within each strain, the dots are measured data, and the lines are the best fit of the model that includes resource-dependent growth and density-dependent growth (Eq (2) in the manuscript). Each replicate has a different color. The Spearman correlation and p-values, together with the 95% confidence levels of the kinetic parameters for each replicate are shown in S5 Data. Most of the samples have a Spearman correlation higher than 0.85 with a p-value that is lower than 0.05. (TIF) [file pcbi.1010565.s012.tif]

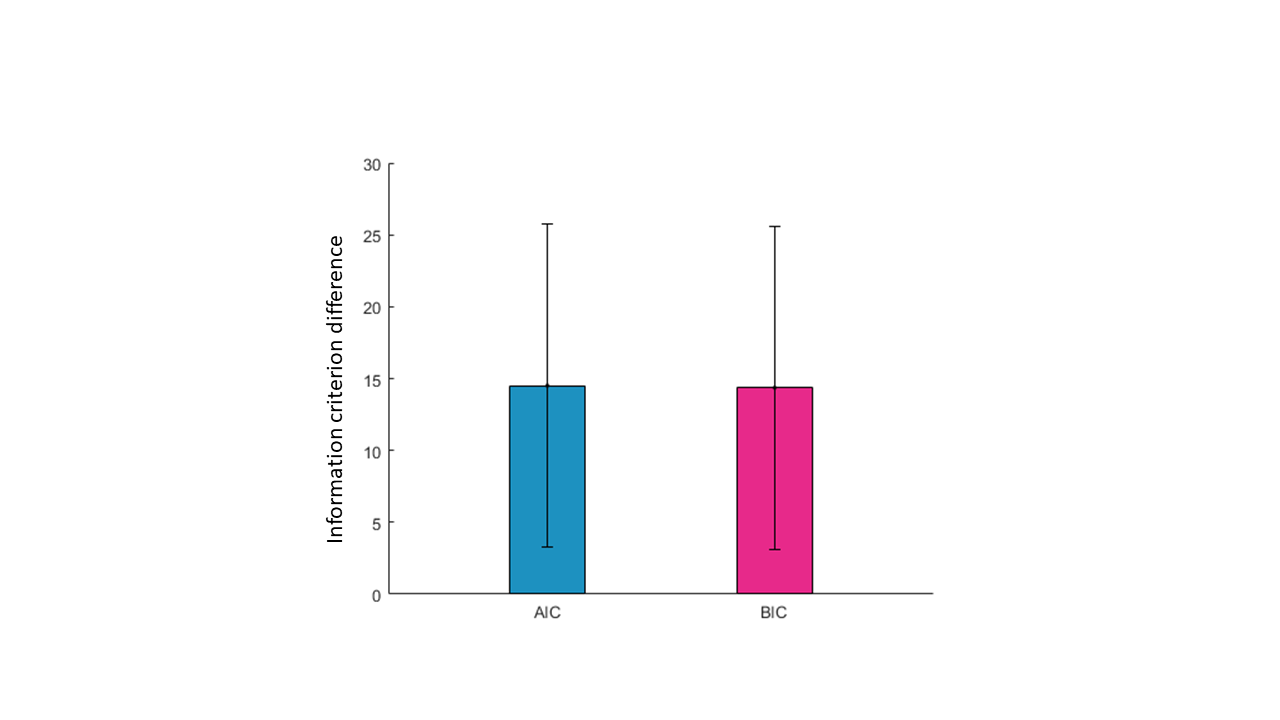

Supplement: S6 Fig — Mean and standard deviation of the difference between the information criterion of the models with or without density-dependent term over all samples. P-values are 2.21×10^−4^ and 4.79×10^−4^ for AIC and BIC, respectively, using Kolmogorov-Smirnov test. (TIF) [file pcbi.1010565.s013.tif]

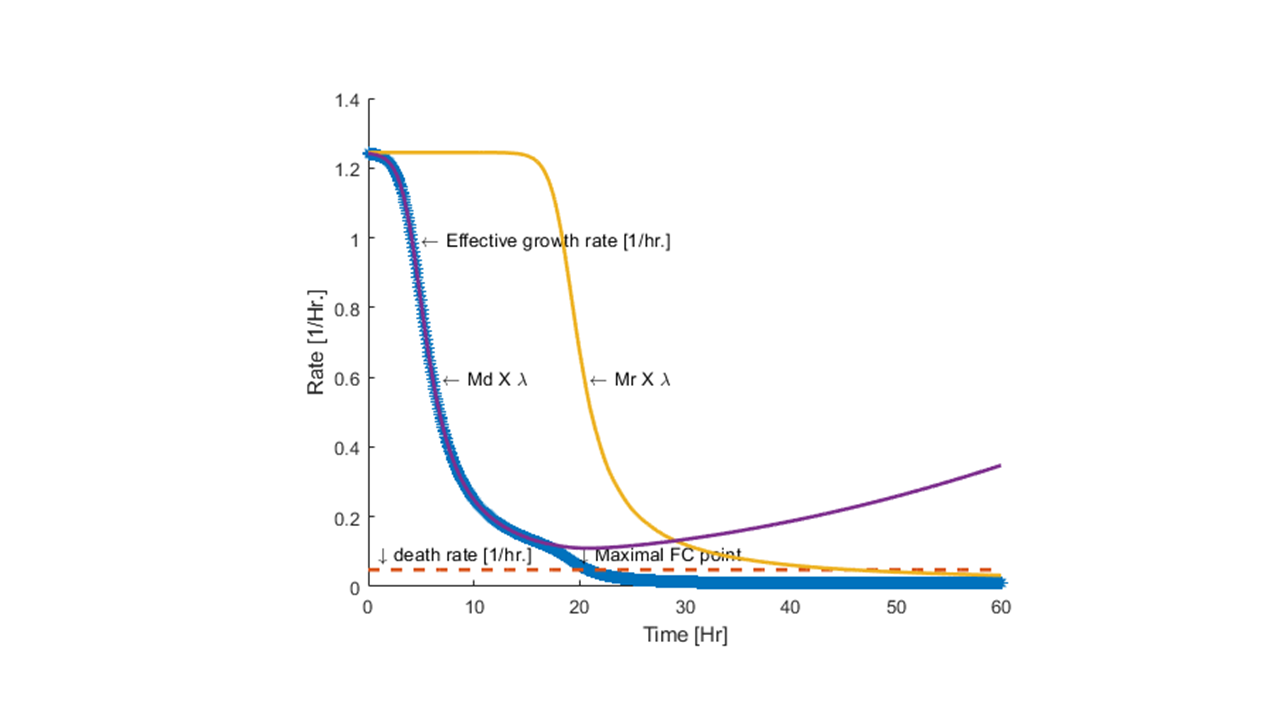

Supplement: S7 Fig — A typical realization of the change in growth terms with time. The growth terms that depend on density (purple) and on resource decline (yellow) with time. The density-dependent term affects first and is responsible for the initial, slow decline in the overall growth rate. The resource limitation term declines fast, as expected. Both growth terms affect the growth in the range where the death term is negligible. (TIF) [file pcbi.1010565.s014.tif]

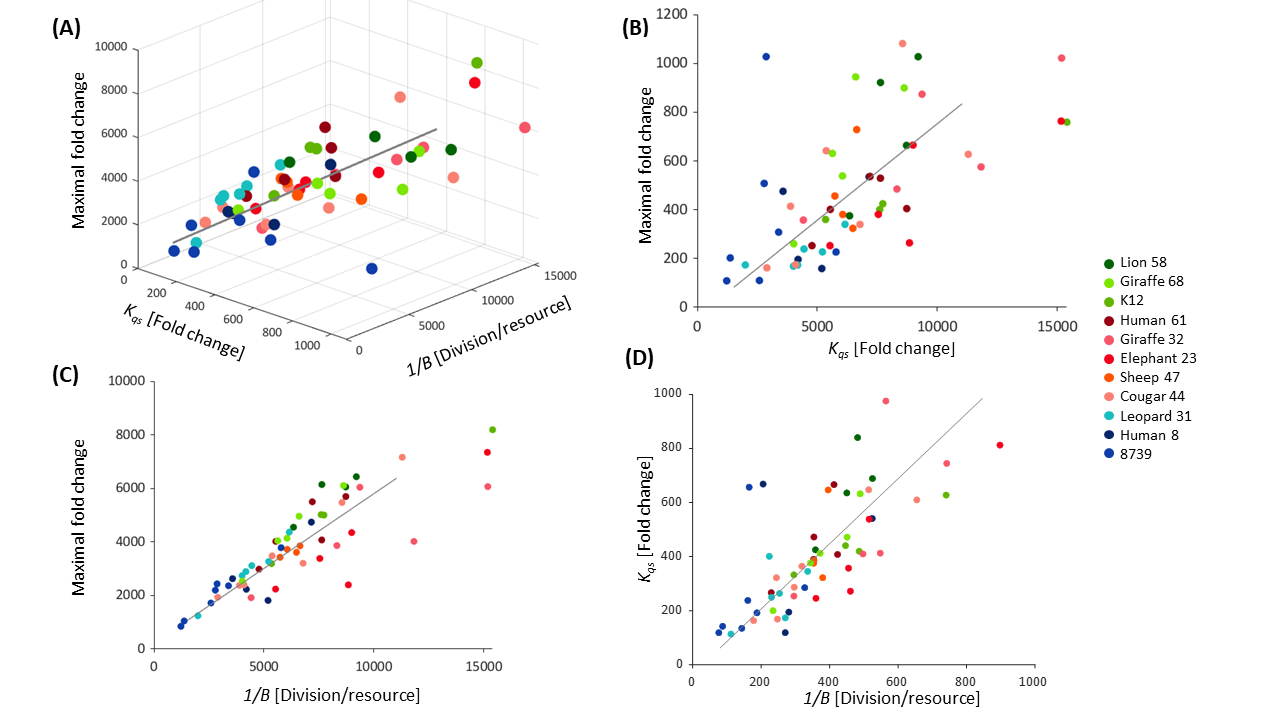

Supplement: S8 Fig — (A) Maximal FC vs. Kqs and 1/B. The dots are the parameters of individual measurement, the color denotes the strain. The black lines are the first principal component and its projections. (B) Maximal FC vs. Kqs. The gray line is the projection of the 3D first principal component. (Pearson: ρ = 0.71; p-value<0.01). (C) Maximal FC vs. 1/B (resource utilization efficiency). The gray line is the projection of the 3D first principal component, (Pearson: ρ = 0.86; p-value <0.01). (D) Kqs vs.1/B. The gray line is the projection of the 3D first principal component, (Pearson: ρ = 0.602; p-value <0.01). (TIF) [file pcbi.1010565.s015.tif]
